# Factors Associated with HPV Vaccine Uptake in College Students Following the COVID-19 Pandemic

**DOI:** 10.3390/vaccines14020122

**Published:** 2026-01-27

**Authors:** Kathleen H. Scarbrough, Sana Malik, Devika Patel, Kiersten Pflueger, Linda Mermelstein, Yunhan Liao, Barbara Nemesure

**Affiliations:** 1 Department of Family, Population and Preventive Medicine, Stony Brook University, Stony Brook, NY 11794, USA; yunhan.liao@stonybrookmedicine.edu (Y.L.); barbara.nemesure@stonybrookmedicine.edu (B.N.); 2 Department of Obstetrics, Gynecology, and Reproductive Medicine, Stony Brook University, Stony Brook, NY 11794, USA; 3 School of Social Welfare, Stony Brook University, Stony Brook, NY 11794, USA; sana.k.malik@stonybrook.edu; 4 Department of Psychology, Stony Brook University, Stony Brook, NY 11794, USA; devika.patel@stonybrook.edu (D.P.); kiersten.pflueger@stonybrook.edu (K.P.); 5 Stony Brook Cancer Center, Stony Brook Medicine, Stony Brook, NY 11794, USA; linda.mermelstein@stonybrookmedicine.edu

**Keywords:** HPV vaccine uptake, college students, COVID-19 pandemic, provider recommendation, health communication, health literacy, prevention

## Abstract

Background: Most cancers caused by human papillomavirus (HPV) are preventable through vaccination, yet uptake among U.S. college students remains below national targets. This study examined HPV vaccination rates and factors associated with vaccine uptake among students aged 18–26 years at a large, diverse public university in New York State following the COVID-19 pandemic. Methods: In March 2022, an online survey was distributed to 19,351 students aged 18–26 years; responses were received from 708 students (~4%) and included in the analysis. Descriptive statistics and multivariable logistic regression was used to identify predictors of HPV vaccination. Results: Overall, 59% of students reported receiving at least one HPV vaccine dose, while 17.7% were unsure of their vaccination status. Among students whose healthcare provider recommended the HPV vaccine, 76.4% were vaccinated compared to 16.7% without one (*p* < 0.001). Healthcare provider recommendation was the strongest predictor of vaccination (OR 17.9; 95% CI: 8.45–37.91). Additional factors significantly associated with uptake included agreement that the HPV vaccine is safe (OR 2.56; 95% CI: 1.54–4.27), importance of a sexual partner being vaccinated (OR 2.65; 95% CI: 1.90–3.69), and valuing family opinion (OR 1.67; 95% CI: 1.23–2.26). Students most preferred receiving HPV information from healthcare providers (73.4%), followed by Internet searches (51.8%) and social media (35.1%). Conclusions: HPV vaccination uptake among college students remains below national targets. Strengthening provider recommendations, addressing safety concerns, and implementing multimodal education strategies during preventive visits for young adults are essential to improve coverage and reduce HPV-related cancer risk.

## 1. Introduction

Human papillomavirus (HPV) is the most common sexually transmitted infection in the United States (U.S.), with an estimated prevalence of 42 million cases in 2024 [1]. Many HPV infections resolve on their own within 1 to 2 years; however, infections that persist increase a person’s risk of developing cervical, anal, vaginal, vulvar, penile, and oropharyngeal cancer [2]. The high-risk or oncogenic HPV subtypes are responsible for 37,000 of these cancers each year within the United States (U.S.) [3]. HPV vaccination is one of the most effective methods to prevent HPV-related cancers, which the Centers for Disease Control and Prevention (CDC) estimates can prevent more than 90% of these cancers [4].

The first HPV vaccination was approved by the US Food and Drug Administration in 2006 for use in 9–26-year-old females [5]. In 2011, the recommendation was expanded to include males aged 9–21 years of age, and in 2019, the recommendation was further expanded for all people aged 9–26 and for those aged 27–45 years based on risk factors [6,7]. Since the HPV vaccination was introduced in the U.S., infections with HPV types that cause most HPV cancers and genital warts have decreased by 88% among teenage girls and 81% among young adult women [4]. Despite the high safety and efficacy of the HPV vaccination in preventing the development of HPV-related cancers, the current uptake of vaccination coverage falls short of the Healthy People 2030 goal of 80% [2]. Only 58.5% of adolescents aged 13–15 years had received at least 2 doses of the HPV vaccine in 2021, with 60% of females and 57.1% of males in this age group being adequately vaccinated [2].

HPV infection and related diseases are significant health issues facing college students, given the nationally low HPV vaccination uptake and their increased rates of sexual activity. Studies have shown that some barriers among college students to receiving the HPV vaccine include a lack of provider recommendation, low levels of knowledge about HPV and the HPV vaccine, and decreased perceived susceptibility of contracting HPV [8,9]. Males have been shown in studies to have lower levels of knowledge about HPV and the HPV vaccine compared to females [8,9,10].

Most studies to date that have evaluated HPV vaccines on U.S. college campuses were conducted before the latest 2019 HPV vaccine guidelines, and primarily included campuses in the Southeast, California, and the Midwest. This study aims to estimate the current HPV vaccine rates among students aged 18–26 years and evaluate factors associated with HPV vaccination status at a large, diverse public university in the Northeast post the COVID-19 pandemic and after the 2019 Advisory Committee on Immunization Practices (ACIP) guidelines.

## 2. Materials and Methods

Stony Brook University (SBU) is located in Suffolk County, New York. For the academic year of 2021–2022, the total student body of 26,608 was composed of 17,999 undergraduates and 8609 graduate students [11]. The university contains 12 schools and offers over 200 degrees and programs to a diverse student body. Women represent 51% of the student body, with 32.6% of students identifying as White, 24.6% Asian, 5.8% Black, 12.6% Hispanic, 2.5% Bi/Multiracial, and 8.5% unknown race, ethnicity [11,12]. Approximately 13.5% of students are international, and 6.5% report a primary residence located out of state, with the majority attending SBU from New Jersey, California, or Connecticut [11,12]. The number of students aged 18–26 years in the spring semester at SBU was 19.351.

A cross-sectional observational study was designed to assess the current rates, perceptions, healthcare provider (HCP) recommendations, and factors that may be associated with HPV, COVID-19, and Flu vaccines among college students [13]. A survey was distributed via email in March 2022 by SBU’s Student Health Services to all enrolled students who were 18–26 years of age. To improve participation, the survey was distributed on a Monday and a Tuesday based on internal reporting data provided by Student Health Services, indicating that students are more likely to open emails sent on these days. The email was sent by the director of Student Affairs to elevate the status of the correspondence and the likelihood that the students would open the email. The timing was chosen to avoid midterm exam periods and school breaks to maximize participation. All participants provided informed consent online before completing the survey. A second reminder email was sent one week after the initial email. For students who chose to complete the survey, a five-dollar gift card was offered as an incentive. This report presents findings specifically related to HPV vaccination, including evaluations of demographic information, HPV and HPV vaccine knowledge, HPV vaccine status, HCP recommendation, student perceptions, and potential barriers/facilitators to HPV vaccination.

The main outcome variable was receipt of at least 1 HPV vaccine (yes, no, not sure). Independent variables included age (years) and age group (18–21 years old, 22–26 years old), race (White, Asian, Black, bi/multiracial, other), birth sex (male/female), ethnicity (Hispanic yes/no), marital status/long-term relationship (yes/no), sexual orientation (heterosexual, homosexual, bisexual, other), HCP recommendation for the HPV vaccine (yes, no, I don’t know). HCP recommendation was assessed using the question, “Has a healthcare provider ever recommended the HPV vaccine for you?” Students were also asked whether they had completed the vaccine series (yes, no, I don’t know). HPV-related knowledge was assessed using 22 items from the HPV knowledge and 10 items from the HPV vaccine knowledge scales (yes, no, I don’t know) originally developed by Waller et al. and later extended and validated by Pérez et al. [14,15]. These items assessed knowledge of HPV transmission, HPV-related cancer risk, vaccine indications, dosing and effectiveness consistent with prior validated instruments. The survey question related to who should receive the vaccine was changed from the original Perez et al. to reflect the CDC recommendation that both males and females receive the HPV vaccine and not the Canadian recommendations [15].

The importance of family opinion, peer opinion, the ability of the vaccine to protect oneself, and the ability of the vaccine to protect their sexual partner when considering receiving the HPV vaccine was assessed using a 5-point Likert scale (not at all important, somewhat important, moderately important, very important, and extremely important). A compressed 3-point scale, including less important (not at all important and somewhat important), moderately important, and more important (very important and extremely important) categories, was further evaluated. Participants were also asked how strongly they agreed with the following statements: “The HPV vaccine is safe” and “I am at risk for getting HPV” were assessed using a 5-point Likert scale (strongly disagree, somewhat disagree, neither agree nor disagree, somewhat agree, strongly agree). These responses were compressed into three categories: disagree (strongly disagree and somewhat disagree), neutral (neither disagree nor agree), and agree (somewhat disagree and strongly agree). All analyses from the 5-point vs. 3-point scales were consistent; therefore, we present the condensed 3-point findings for ease of interpretation.

### Statistical Analysis

Of the 19,351 surveys that were distributed, 797 (~4%) were completed. Three duplicate surveys were excluded, and 708 were analyzed using an 80% completion cut-off. Of those, 100% provided a response to the HPV vaccine status question. Descriptive statistics (N and %) are presented to provide data related to the distribution of student demographic characteristics, perceptions, and knowledge about HPV and HPV vaccination. Pearson chi-square and Welch’s *t*-test were performed to evaluate differences between groups. Variables with *p* < 0.05 in the univariate analyses were considered statistically significant and were included in subsequent multivariable logistic regression models. Results are presented as odds ratios (ORs) and 95% confidence intervals (CIs). Multivariable logistic regression models retained the original response categories given their conceptual relevance. Model convergence and separation diagnostics were assessed, with no evidence of complete or quasi-complete separation. Observations with missing covariate data were excluded from multivariate models (complete-case analysis). Missingness was low (<5%) and distributed across multiple variables, with no more than eight missing responses per variable. Statistical analysis was performed using SAS version 9.4.

The Stony Brook University Institutional Review Board (IRB) approved this study (IRB #2021-00479), and the guidelines in the Declaration of Helsinki were upheld.

## 3. Results

The mean age of the 708 student participants was 21.08 (SD 2.2) years, with 304 (44.4%) identifying as White, 287 (41.9%) as Asian, 36 (5.3%) as Bi/Multiracial, 25 (3.7%) as Black, and 10.7% as Hispanic. The majority of students were undergraduates (541/708, 76.4%), heterosexual (511/708, 75.5%), and were born in the United States (572/708, 80.9%). The overall rate of students receiving at least 1 HPV vaccine was 59% (418/708), and 17.7% (125/708) were unsure of their vaccine status. Of the students starting the HPV series, 86.6% report completing the series (362/418). Because relatively few students reported non-completion and some were unsure about series completion, we did not examine predictors of completion status, as subgroup analyses would have been underpowered and difficult to interpret.

Table 1 presents student characteristics stratified by HPV vaccine status. The rates of receiving at least one HPV vaccine were 62.4% (332/532) for females compared to 49.1% (84/171) for males (*p* = 0.006). Students who were in a long-term relationship or married had higher rates of at least one HPV vaccination and lower rates of being unsure of their vaccine status compared to those who were not married or in a long-term relationship (*p* = 0.02). For students who had ever received a HCP recommendation to receive the HPV vaccine, 76.4% (391/512) reported receiving the vaccine compared to 16.7% (16/96) who did not receive a recommendation and 11% (11/100) who were unsure whether they had ever received such a recommendation (*p* < 0.001). There were no significant differences in HPV vaccination rates by age, race, ethnicity, religion, sexual orientation, or having medical insurance as a child or teenager.

Student perceptions about HPV vaccines and potential barriers/facilitators to vaccination are presented in Table 2. Students who agreed that the HPV vaccine was safe had a vaccination rate of 70.1% (364/519) compared to 22.2% (4/18) for those who disagreed and 29.1% (46/158) for those who were neutral about vaccine safety (*p* < 0.001). Placing more value on peer opinion and belief in the importance of their sexual partner being vaccinated resulted in an increased likelihood of receiving at least one HPV vaccine (*p* < 0.001). Higher rates of HPV vaccination were also seen among students who felt it was important that the vaccine could protect them against HPV (*p* = 0.01), and among those who agreed that they were at risk for getting HPV (*p* = 0.06). The importance of the vaccine to protect their partner did not significantly impact vaccine status.

Differences between male and female participants regarding HPV vaccination are presented in Table 3. The rate of provider recommendation to receive the HPV vaccination was lower for males (102/171, 59.6%) compared to females (391/512, 76.5%; *p* < 0.001). The mean scores on both the HPV knowledge and HPV vaccine knowledge tools were lower for male compared to female students (*p* = 0.006 and *p* < 0.001, respectively). Item-level responses further revealed specific knowledge gaps; while most students recognized the association between HPV and cervical cancer, substantially fewer were aware of its association with anal or oral cancers.

Table 4 presents the logistic regression models for HPV vaccine status (yes vs. no) by factors found to be significant in the univariable analysis. The findings indicated that provider recommendation (OR 17.9, (95% CI 8.45, 37.91)), agreement that HPV vaccine is safe (OR 2.56, (CI 1.54, 4.27)), agreement with the importance of partner being vaccinated against HPV (OR 2.65, (CI 1.9, 3.69)), and agreement with the importance of family opinion when considering HPV vaccination (OR 1.67, (CI 1.23, 2.26)) were significantly associated with students receiving at least one HPV vaccination. Note that wider confidence intervals for certain subgroup-specific odds ratio estimates, such as those for “Any friends receive the HPV vaccine” and “Provider recommendation”, were attributable to small proportion of respondents in the “No” category.

Students who responded that they did not receive any HPV vaccine (n = 165) were asked about factors that contributed to their decision not to receive the HPV vaccine. The questionnaire allowed for multiple responses to this item. The most common reason provided was not knowing why they were unvaccinated (36.9%), followed by cost of the vaccine (28.1%), never being offered the vaccine (27.5%), and concern about vaccine safety (25.0%).

All participants were asked how they would prefer to receive information about HPV and HPV vaccines, again allowing for multiple responses. Healthcare provider was the most preferred method with 73.4% of the students selecting this option, followed by Internet/Google search (51.8%) and social media (35.1%). Other communication methods that ranked lower in preference were peer educators at campus events (24.3%), information boards posted throughout campus (21.5%), healthcare apps (19.6%), and pamphlets/mailings (18.8%).

## 4. Discussion

This study estimated HPV vaccination rates and identified factors associated with vaccine uptake among a diverse student population at a large public university in New York, a state with some of the highest adolescent HPV vaccination rates in the United States. Even in this favorable setting, only 59% of students reported receiving at least one HPV vaccine dose—well below the Healthy People 2030 target of 80% [16] and far from optimal for a population at risk for HPV-related cancers. Nearly 40% of male and 25% of female students reported never receiving a HCP recommendation for HPV vaccination, despite strong evidence that such recommendations are a primary driver of vaccine uptake. Study findings indicated that those students with a provider recommendation were 17 times more likely to have initiated the HPV vaccine series than those without this recommendation. Lower HPV vaccine rates were reported among males and students who were single. There were no significant differences in HPV vaccination rates by age, race/ethnicity, religion, sexual orientation, or having medical insurance as a child/teen.

The overall HPV vaccination rate observed in this study falls in the range reported in recent studies of college populations, which have documented vaccination rates from 30% to 77% depending on the setting and sample characteristics [17,18,19]. Consistent with prior research, approximately one in five students in this study was unsure of their vaccination status [17], suggesting low personal awareness of vaccine history. While colleges often require documentation of select immunizations (e.g., MMR and, in some cases, meningococcal vaccines such as MenB) at matriculation, comprehensive vaccination histories such as HPV are not consistently reviewed or emphasized, which may contribute to limited student awareness of HPV vaccination status. While national adolescent vaccination rates have increased steadily, uptake among college-aged populations appears to have plateaued in recent years [20], potentially due to disruptions in preventive health services during the COVID-19 pandemic [21]. These disruptions may have included reallocation of public health resources, temporary clinic closures, shifts to telehealth, and increased hesitancy in seeking in-person care. Comparisons with prior research highlight variability in vaccination rates. For example, Kitur et al. (2021) reported a 63.4% rate among undergraduates, whereas Goldfarb et al. (2022) found a 77.8% rate at a private university [17,18]. These differences may reflect variations in demographics, institutional resources, and access to healthcare. Our population included a substantially higher proportion of Asian students and a lower proportion of White, Black, and multiracial students than these earlier reports. Other studies, such as the one reported by Lin et al. (2023), indicated lower vaccination rates for both males (36%) and females (53%) compared to findings from the present investigation, thereby suggesting possible geographic or institutional influences [19].

Consistent with previous research, males in this study had lower vaccination rates, HPV knowledge, and HPV vaccine knowledge, and were less likely to receive a provider recommendation compared with females [8,10]. In 2018, HPV and HPV vaccine knowledge were moderately understood among college students [17], with results from the current 2022 survey indicating little change since before the pandemic. In 2022, HPV knowledge scores among college students decreased from scores in 2019 [22]. Many male college students remain unaware of the modes of transmission, the asymptomatic nature of HPV, the consequences, and the risk factors for HPV [23,24]. Male knowledge scores in the current sample followed a similar pattern, as they were significantly lower than those of females. The gap between male and female vaccine rates found in this study is consistent with previous reports, though the margin appears to be narrowing [25]. This outcome persists despite the medical community’s awareness of the gap and multiple communication strategies by the vaccine manufacturer, public health departments, and college-level initiatives to improve the rates, especially among males [26]. According to the National College Health Assessment-II, in 2013, 49.5% of males aged 18–21 years and 28.6% of males aged 22–26 years received the HPV vaccine, compared to 71.2% of females aged 18–21 years and 62.2% of females aged 22–26 years [27]. The present study found higher overall rates for males and females of HPV vaccination compared to the 2013 study and indicated that there is no longer a significant difference between the 18–21- and 22–26-year-old age groups (Table 1). A recent nationwide survey revealed that only 53% of college students—about 41% of men and 57% of women—were up to date with the recommended HPV vaccination in 2022 [28]. In 2018, only 54% of women and 27% of men ages 18–26 years had initiated the vaccine series, with only 22% of young adults completing the series [29]. These gaps underscore the need for targeted interventions given the substantial burden of HPV-related cancers. Future studies could explore item-level gender differences in HPV and HPV vaccine knowledge and further characterize unvaccinated subgroups to guide targeted interventions.

Healthcare provider recommendation was the strongest predictor of HPV vaccine initiation in this study. Students who received a HCP recommendation had 4.5 times higher HPV vaccine uptake than those without a HCP recommendation. This finding is consistent with prior studies and highlights the influential role of healthcare providers in vaccine decision-making. However, many college students do not have regular contact with healthcare providers, particularly if they do not utilize student health services, suggesting that strategies relying solely on provider interactions may fail to reach a large portion of the population. These findings highlight the need for structured preventive care visits during late adolescence and early childhood (around age 18–21 years) to support transition from parent-managed care to self-managed care. Such visits should include a comprehensive review of vaccination history at the time of college entry or other routine points of healthcare contact in early adulthood, counseling on catch-up vaccines, and education on accessing personal health records.

Perceptions of vaccine safety, the importance of family opinion, and valuing partner vaccination were all associated with higher uptake. However, knowledge gaps remain considerable. While most students recognized that HPV could cause cervical cancer, fewer than one in four were aware of its link to anal or oral cancers. Notably, knowledge scores were not significantly associated with vaccine uptake in regression models, suggesting that awareness alone may be insufficient without a HCP recommendation or other enabling factors. Among unvaccinated students, the most common reasons cited were uncertainty about why they were unvaccinated, cost factors, never being offered the vaccine, and safety concerns.

Health literacy emerged as a critical barrier. One in five students did not know their HPV vaccination status, and among those who reported being unvaccinated, over one-third (36%) were unsure why they had not received the vaccine. This uncertainty reflects a gap in students’ ability to access, understand, and/or use their personal health information—skills that are essential for informed decision-making. Limited health literacy in this context may reduce students’ capacity to seek vaccination proactively, even when access barriers are minimal. Interventions should prioritize record access (patient portals, state immunization information systems), HCP communication, and tailored education to empower young adults. Further characterization of students who were unsure of their HPV vaccination status represents an important area for future research.

One possible explanation for why approximately one in five students report not having received a vaccine recommendation or do not know their vaccination status is that HPV vaccination is typically initiated between ages 9 and 12, with counseling directed primarily to parents rather than the child. The CDC recommends routine HPV vaccination at ages 11–12, although initiation can begin as early as age 9, a strategy increasingly promoted to improve completion rates and cancer prevention outcomes [30]. At these younger ages, adolescents are rarely the primary recipients of vaccine-related information, and even if included in discussions, they may not recall these details as young adults. As individuals transition to adulthood, expert guidelines emphasize the importance of comprehensive preventive care visits around ages 18–21, which should include a review of vaccination history, timing of future boosters, sexual health counseling, and other lifestyle recommendations [31]. This study’s findings support the need for a structured primary care visit during this transition period to reinforce vaccine awareness and address gaps in knowledge. Such visits could reduce the high prevalence of young adults who are unaware of their own vaccination status and improve adherence to preventive health recommendations.

Students’ strong preference for healthcare providers as an information source (73%), combined with secondary reliance on Internet searches (52%) and social media (35%), underscores the need for multimodal communication strategies. Campus health services should leverage these preferences by pairing provider counseling with accessible digital content. Our ongoing work testing educational materials in student focus groups reflects the importance of tailoring interventions to young adults’ preferred learning channels.

The college setting offers a valuable opportunity to vaccinate young adults who were missed in adolescence. Barriers common in younger populations—such as lack of insurance, limited clinic access, or parental hesitancy—are often reduced in this environment. Additionally, one-third of students in this study had never engaged in sexual activity, making them ideal candidates for vaccination before potential HPV exposure. Vaccination remains beneficial even for those who are sexually active [30]. Our findings also highlight students’ preferred sources of HPV vaccine information and are consistent with findings of the focus groups held with a subgroup of these participants [13]. While healthcare providers remain the most trusted source, students also cited internet searches and social media, suggesting that a multimodal communication approach may be most effective. Campus health services can implement multimodal strategies—HCP reminders, standing orders, and digital outreach to normalize HPV vaccination as part of routine preventive care. Overall, these findings reinforce the importance of transition-focused preventive care and health literacy interventions to improve HPV vaccine uptake among young adults.

Future research should build on these findings by examining item-level HPV and HPV vaccine knowledge difference by gender, further characterizing unvaccinated students and those uncertain of their status, and evaluating interventions that enhance HCP recommendation and health literacy during the transition to adulthood. Studies incorporating multi-institutional samples, verified vaccine records, and prospective designs may further clarify barriers to HPV vaccine uptake and inform scalable, targeted interventions for college-aged populations.

### Limitations

Although this study is strengthened by its large sample size and highly relevant target population, it has several limitations. First, the study design was cross-sectional and relied on self-reported data, which may be subject to recall bias, particularly regarding vaccination status. Second, although the sample was diverse, it reflects the demographics of a single public university and may not be generalizable to all college populations. Finally, vaccination status was not verified through medical records. Despite these limitations, the findings highlight important knowledge gaps and challenges related to HPV and HPV vaccination, and factors may inform the development of educational initiatives to increase awareness in this population.

An additional limitation is the low response rate, (~4%) which raises the possibility of non-response bias. Students who chose to participate may differ from non-respondents, including having greater interest in health-related topics or prior HPV vaccination. In addition, the respondent sample overrepresented White and Asian students relative to the overall university population, which may further limit generalizability. While this limitation may affect estimates of HPV vaccination prevalence, the primary focus of this study was to examine associations between HPV vaccination and factors such as healthcare provider recommendation and vaccine-related perceptions. These associations are consistent with prior research and are likely to remain informative despite potential response bias. Although individual-level demographics of non-responders were not available, overall student demographic characteristics of the university are described in the Methods to provide institutional context; accordingly, findings should be interpreted as reflecting the perspectives of respondents rather than the entire student population. Additionally, because HPV vaccination guidelines and clinical practice environments evolve over time, the applicability of our findings to future college populations may change.

## 5. Conclusions

This study confirms that although HPV vaccination uptake has increased among college students aged 18–26 years, it remains well below the target goal of 80%, even at a diverse public university in the Northeast, where HPV vaccination uptake is notably higher than national averages. The findings of low health literacy, suboptimal provider recommendation rates for HPV vaccination, and concerns about vaccine safety should be key focal points of future interventions targeting this population.

## Figures and Tables

**Table 1:** Characteristics of students by HPV vaccination status (at least one vaccine dose).

Variable	No. of Students (%)				*p*- Value
	Total (n = 708)	Yes (n = 418)	No (n = 165)	Not Sure (n = 125)	
**Birth Sex**, n (%)					**0.01**
Female	532 (75.7)	332 (62.4)	111 (20.9)	89 (16.7)	
Male	171 (24.3)	84 (49.1)	52 (30.4)	35 (20.5)	
**Age, y, mean (SD)**	21.08 (2.2)	21.08 (2.17)	21.32 (2.35)	20.77 (2.07)	0.11
**Age group, y**					0.11
18–21	436 (63.9)	256 (58.7)	93 (21.3)	87 (20)	
22–26	246 (36.1)	150 (61)	62 (25.2)	34 (13.8)	
**Race**, n (%)					0.63
White	304 (44.4)	187 (61.5)	71 (23.4)	46 (15.1)	
Asian	287 (41.9)	161 (56.1)	65 (22.7)	61 (21.2)	
Black	25 (3.6)	15 (60)	5 (20)	5 (20)	
Other	33 (4.8)	22 (66.7)	7 (21.2)	4 (12.1)	
Bi/Multiracial	36 (5.3)	23 (63.9)	9 (25)	4 (11.1)	
**Hispanic**, n (%)					0.61
Yes	74 (10.7)	45 (60.8)	19 (25.7)	10 (13.5)	
No	618 (89.3)	366 (59.2)	141 (22.8)	111 (18)	
**Religion**, n (%)					0.05
Buddhist	29 (4.1)	19 (65.5)	4 (13.8)	6 (20.7)	
Christian	213 (30.5)	127 (59.6)	45 (21.1)	41 (19.2)	
Jewish	38 (5.4)	27 (71.1)	5 (13.2)	6 (15.8)	
Hindu	42 (6.0)	18 (42.9)	17 (40.5)	7 (16.7)	
Muslim	52 (7.4)	31 (59.6)	12 (23.1)	9 (17.3)	
Sikh	10 (1.4)	3 (30.0)	2 (20.0)	5 (50.0)	
Higher power but not a religion	72 (10.3)	47 (65.7)	19 (26.4)	6 (8.3)	
Other	48 (6.9)	27 (56.3)	15 (31.3)	6 (12.5)	
Atheist/Agnostic	195 (27.9)	117 (60.0)	44 (22.6)	34 (17.4)	
**Sexual Orientation**, n (%)					0.22
Heterosexual	511 (75.5)	289 (56.6)	122 (23.9)	100 (19.6)	
Homosexual	33 (4.9)	19 (57.6)	9 (27.3)	5 (15.2)	
Bisexual	109 (16.1)	74 (67.9)	23 (21.1)	12 (11)	
Other	24 (3.6)	17 (70.8)	5 (20.8)	2 (8.3)	
**Transgender**, n (%)					0.37
Yes	11 (1.6)	8 (72.7)	3 (27.3)	0 (0)	
No	681 (98.4)	398 (58.4)	160 (23.5)	123 (18.1)	
**Born in the U.S.**, n (%)					0.09
Yes	572 (80.9)	345 (60.3)	123 (21.5)	104 (18.2)	
No	135 (19.1)	73 (54.1)	41 (30.4)	21 (15.6)	
**Student type**, n (%)					0.53
Undergraduate	541 (76.4)	318 (58.8)	123 (22.7)	100 (18.5)	
Graduate	167 (23.6)	100 (59.9)	42 (25.2)	25 (15)	
**Married/Long term relationship**, n (%)					**0.01**
Yes	225 (31.8)	146 (64.9)	53 (23.6)	26 (11.6)	
No	482 (68.2)	272 (56.4)	112 (23.2)	98 (20.3)	
**Insurance as a child/teen**, n (%)					0.13
Yes	579 (82.8)	336 (58.0)	135 (23.3)	108 (18.6)	
No	120 (17.2)	80 (66.7)	26 (21.7)	14 (11.7)	
**Sexual activity, ever**, n (%)					**0.02**
Yes	403 (58.0)	246 (61.0)	102 (25.3)	55 (13.6)	
No	239 (34.4)	140 (58.6)	48 (20.1)	51 (21.3)	
Prefer not to answer	53 (7.6)	28 (52.8)	10 (18.9)	15 (28.3)	
**Friends have received HPV vaccine**, n (%)					**<0.001**
Yes	295 (42.2)	243 (82.4)	46 (15.6)	6 (2.0)	
No	20 (2.9)	4 (20.0)	11 (55.0)	5 (25.0)	
I don’t know	384 (54.9)	168 (43.8)	106 (27.6)	110 (28.7)	
**Romantic partner has received HPV vaccine**, n (%)					**<0.001**
Yes	88 (12.6)	74 (84.1)	13 (14.8)	1 (1.1)	
No	57 (8.2)	22 (38.6)	29 (50.9)	6 (10.5)	
I don’t know	187 (26.8)	105 (56.2)	47 (25.1)	35 (18.7)	
No romantic partner	367 (52.5)	214 (58.3)	74 (20.2)	79 (21.5)	
**Provider recommendation**, n (%)					**<0.001**
Yes	512 (72.3)	391 (76.4)	89 (17.4)	32 (6.2)	
No	96 (13.6)	16 (16.7)	57 (59.4)	23 (24.0)	
I don’t know	100 (14.1)	11 (11.0)	19 (19.0)	70 (70.0)	

Note: Bolded signifies significant *p*-values.

**Table 2:** Perceptions and knowledge of students stratified by HPV vaccination.

Variable	No. of Students (%)				*p*- Value
	Total (N = 708)	Yes (n = 418)	No (n = 165)	Not Sure (n = 125)	
Agreement with the following statements:					
Importance of (when considering HPV vaccination):					
**Family Opinion**, n (%)					0.05
Less important	282 (40.5)	153 (54.3)	80 (28.4)	49 (17.4)	
Moderately important	219 (31.4)	131 (59.8)	49 (22.4)	39 (17.8)	
More Important	196 (28.1)	130 (66.3)	33 (16.8)	33 (16.8)	
**Peer opinion**, n (%)					**<0.001**
Less important	386 (55.5)	221 (57.3)	101 (26.2)	64 (16.6)	
Moderately important	199 (28.6)	109 (54.8)	43 (21.6)	47 (23.6)	
More Important	110 (15.8)	83 (75.5)	18 (16.4)	9 (8.2)	
**Ability of vaccine to protect me**, n (%)					**0.01**
Less important	24 (3.4)	9 (37.5)	9 (37.5)	6 (25.0)	
Moderately important	74 (10.6)	33 (44.6)	24 (32.4)	17 (23.0)	
More Important	599 (86.0)	372 (62.1)	129 (21.5)	98 (16.4)	
**Ability of vaccine to protect partner**, n (%)					0.10
Less important	41 (5.9)	24 (58.5)	10 (24.4)	7 (17.0)	
Moderately important	80 (11.5)	37 (46.3)	22 (27.5)	21 (26.2)	
More Important	574 (82.6)	352 (61.3)	130 (22.7)	92 (16.0)	
**My partner being vaccinated against HPV**, n (%)					**<0.001**
Less important	96 (13.8)	28 (29.2)	46 (47.9)	22 (22.9)	
Moderately important	192 (27.6)	96 (50.0)	50 (26.0)	46 (24.0)	
More Important	407 (58.6)	290 (71.2)	65 (16.0)	52 (12.8)	
Knowledge score:	mean (SD)				
HPV ^a^	9.98 (6.66)	11.06 (6.23)	10.07 (7.13)	6.24 (6.09)	**<0.001**
HPV vaccine ^b^	4.75 (3.08)	5.56 (2.72)	4.31 (3.21)	2.57 (2.92)	**<0.001**

^a^ range of potential score is 0–22; ^b^ range of potential score is 0–10; Note: Bolded significant *p*-value.

**Table 3:** HPV/HPV vaccine knowledge and provider recommendation by birth sex.

Variable	Male (N = 171)	Female (N = 532)	*p*-Value
**Provider recommendation**, n (%)			<0.001
Yes	102 (59.6)	407 (76.5)	
No	40 (23.4)	55 (10.3)	
I don’t know	29 (17)	70 (13.2)	
HPV knowledge ^a^, mean (SD)	8.73 (7.15)	10.38 (6.44)	0.01
HPV vaccine knowledge ^b^, mean (SD)	3.84 (3.19)	5.04 (3.00)	<0.001

^a^ range of potential score is 0–22; ^b^ range of potential score is 0–10.

**Table 4:** Logistic regression models for factors associated with at least one HPV vaccine (yes vs. no).

Variable	OR (95% CI)	*p*-Value
**Birth Sex**,		0.89
Male	reference	
Female	0.96 (0.55, 1.68)	
**Born in the U.S.**	1.03 (0.54, 1.96)	0.94
**Any friends receive the HPV vaccine**		0.06
No	reference	
Yes	3.83 (0.82, 17.89)	
Not sure	2.33 (0.51, 10.74)	
**HPV vaccine knowledge**	0.99 (0.91, 1.09)	0.90
**Provider recommendation**		**<0.001**
No	reference	
Yes	17.9 (8.45, 37.91)	
I don’t know	2.23 (0.79, 6.36)	
HPV vaccine is safe ^a^	2.56 (1.54, 4.27)	**<0.001**
Importance of (when considering HPV vaccination):		
**Family opinion**	1.67 (1.23, 2.26)	**<0.001**
**Sexual partner being vaccinated against HPV**	2.65 (1.9, 3.69)	**<0.001**
**Ability of HPV vaccine to protect me**	0.84 (0.5, 1.41)	0.51

^a^ agreement with this statement based 3-scale Likert scale Note: 22 missing values were excluded. Significant *p*-vales bolded.

## Data Availability

The data presented in this study are stored on secure university servers and are subject to Institutional Review Board (IRB) restrictions. Access can be granted upon reasonable request to the corresponding author and with appropriate IRB approval.

## Abbreviations

The following abbreviations are used in this manuscript:

HPV	Human Papilloma Virus
HCP	Healthcare provider
U.S.	United States
CDC	Center for Disease Control and Prevention
ACIP	Advisory Committee on Immunization Practices
SBU	Stony Brook University
